# Interplay of Quantum Confinement and Strain Effects in Type I to Type II Transition in GeSi Core–Shell Nanocrystals

**DOI:** 10.1021/acs.jpcc.2c07024

**Published:** 2023-01-05

**Authors:** Ivan Marri, Simone Grillo, Michele Amato, Stefano Ossicini, Olivia Pulci

**Affiliations:** † Department of Sciences and Methods for Engineering, University of Modena and Reggio Emilia, 42122 Reggio Emilia, Italy; ‡ Interdepartmental Center for Research and Services in the Field of Hydrogen Production, Storage and Use H2 − MO.RE, Via Università 4, 41121 Modena, Italy; ¶ Centro Interdipartimentale En&Tech, 42122 Reggio Emilia, Italy; § Department of Physics, University of Rome Tor Vergata, and INFN, Via della Ricerca Scientifica 1, I-00133 Rome, Italy; ∥ Université Paris-Saclay, CNRS, Laboratoire de Physique des Solides, 91405 Orsay, France; ⊥ Centro S3, Institute of Nanoscience — Italian National Research Council (CNR-NANO), via Campi 213/A, 41125 Modena, Italy

## Abstract

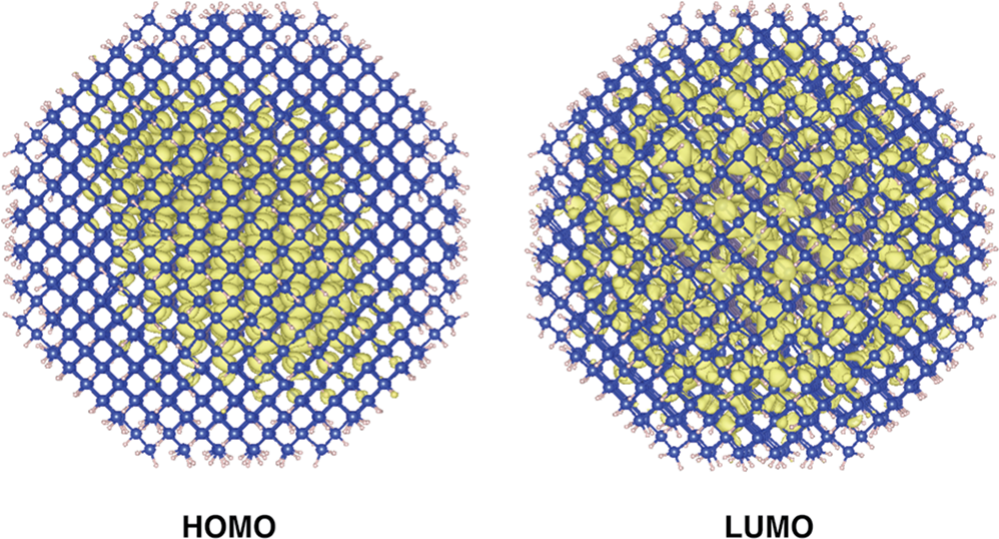

The electronic properties of hydrogenated, spherical SiGe and GeSi core–shell nanocrystals, with a diameter ranging from 1.8 to 4.0 nm, are studied within density functional theory. Effects induced by quantum confinement and strain on the near-band-edge state localization, as well as the band-offset properties between Si and Ge regions, are investigated in detail. On the one hand, we prove that SiGe core–shell nanocrystals always show a type II band-offset alignment, with the HOMO mainly localized on the Ge shell region and the LUMO mainly localized on the Si core region. On the other hand, our results point out that a type II offset cannot be observed in small (diameter less than 3 nm) GeSi core–shell nanocrystals. In these systems, quantum confinement and strain drive the near-band-edge states to be mainly localized on Ge atoms, i.e., in the core region. In larger GeSi core–shell nanocrystals, instead, the formation of a type II offset can be engineered by playing with both core and shell thickness. The factors which determine the band-offset character at the Ge/Si interface are discussed in detail.

## 1 Introduction

The electronic, transport, and optical properties of silicon (Si) and germanium (Ge) nanomaterials have been largely investigated in the past, both experimentally and theoretically, due to their promising applications in optoelectronics and photovoltaics.^1−27^ Moreover, it has been shown that Si and Ge can be combined to obtain innovative materials that can be easily integrated into existing devices. Compared to pure Si and Ge materials, Si/Ge heterostructures offer more possibilities to tune the above-said properties.^28,29^ This can be achieved by varying Si and Ge atom concentration and their spatial disposition, by modifying the geometry of Si/Ge interface, and by modulating both strain and the quantum confinement effect (QCE) to obtain systems with the desired properties.

Si/Ge heterostructures have been fabricated using different techniques, such as molecular beam epitaxy,^30^ self-assembly,^31,32^ ion beam and magnetic sputtering deposition,^33−36^ chemical vapor deposition,^37,38^ chemical synthesis,^39^ and gas-phase and nonthermal plasma synthesis.^40−42^ They have been integrated into different technological devices, for instance, in high-speed and high-power field-effect transistors,^43−47^ photodetectors,^48,49^ linear and nonlinear optics devices,^50,51^ solar cell systems,^52,53^ nonvolatile memory,^54^ and thermoelectric^55^ devices.

Nowadays, the research focused on Si/Ge nanosystems, in particular core–shell (CS) nanowires (NWs)^56−59^ and nanocrystals (NCs),^60−62^ represents one of the most rapidly developing areas in materials science. CS nanosystems offer the possibility of engineering electronic and optical properties, by varying core diameter and shell thickness^63−65^ (thus modulating strain and QC of both core and shell regions) and by switching core and shell materials.

In these systems, electrical and optical response are strongly influenced by the band-edge alignment, which is responsible for the relative localization of electrons and holes; as a consequence, a detailed comprehension of the mechanisms that rule the band-offset character (type I or type II) is of paramount importance. In CS structures, the band offset can result in a type I (band edges localized on the same material) or in a type II (band edges localized on different materials). Type I materials show a strong overlap between electron and hole wave functions, which can be exploited in light-emitting devices. Type II alignment shows, instead, a weaker overlap between electron and hole wave functions. This generally induces longer radiative lifetimes, lower excitation binding energies, and smaller exciton oscillator strengths when compared to type I structures. Leading to a reduction of nonradiative Auger recombination rates—because of the favored photogenerated charge carriers separation and their extraction—type II heterostructures are of great interest for photovoltaics applications.^66^

Recently, we have investigated electronic and optical properties of SiGe and GeSi CSNWs, pointing out how, for instance, the formation of a type II offset can drive the generation of a one-dimensional electron and hole gas.^67^ Here, we focus on the study of SiGe and GeSi CSNCs (in this notation, the first material is in the core, the second in the shell) and, in particular, on the mechanisms that influence the formation of the band-offset between the core and the shell region.

Energy level alignment between Si and Ge bulks reveals a type II offset for Si/Ge superlattice heterostructures, a band-edge profile that, however, can be altered by strain and QC when low dimensionality is taken into account.

Electronic and optical properties of both SiGe and GeSi CSNCs have been discussed in different theoretical works.^63,68−72^ While these works uniquely agree in indicating a strong influence of both core and shell size on electronic and optical gap, as well as the presence of a type II offset in both SiGe and large GeSi CSNCs, they do not agree in (i) defining the character of the band offset and (ii) clarifying the role played by QC and strain when small (diameter less than 4 nm) GeSi CSNCS are taken into account. In this work, we study, within the density functional theory (DFT) framework, SiGe and GeSi CSNCs of different sizes and compositions, and we clarify the mechanisms behind the formation of a type II offset, shedding light on the role played by QC and strain. A particular attention is dedicated to the analysis of the band-offset profile of GeSi CSNCs. The possibility of controlling with accuracy their size and shell thickness (from few nm to tens of nm),^35,41^ together with their high stability, makes these structures more promising than the SiGe CSNCs for technological applications. Noticeably, GeSi CSNCs are more stable than Ge NCs,^73^ and the presence of a Si-capped shell prevents Ge oxidation (and thus the formation of a GeO oxide layer) that would drastically reduce Ge light emission properties and thus its potential applications.

To carry out our investigation, we adopt two different approaches. In the first one, the whole CSNC is taken into account without any simplification. This approach is used to obtain a quantitative signature of the band offset. In the second method, core and shell are analyzed separately, thus adopting an approach that allows to better clarify the role played by both strain and QC.

## 2 Method

The structural and electronic properties of different spherical SiGe and GeSi CSNCs, with diameters ranging from 1.8 to 4.0 nm, have been investigated by means of first-principles calculations. Only hydrogenated NCs have been considered, thus excluding configurations leading to the formation of near-band-edge surface states. This coverage is representative for structures synthesized in strong acidic PH conditions or of systems grown in a hydrogen-rich atmosphere, that is, of NCs characterized by a relevant percentage of hydrogens at the surface.^41,74^ In our approach, SiGe (GeSi) CSNCs are obtained, starting from spherical Ge (Si) NCs, by replacing the Ge (Si) atoms within an internal sphere, centered in the NC, with Si (Ge) atoms. Here, we follow the notation Si_*x*_Ge_*y*_H_*z*_ (Ge_*x*_Si_*y*_H_*z*_) to identify an H_*z*_-terminated spherical CSNC constituted by *x* Si (Ge) atoms located in the core and *y* Ge (Si) atoms located in the shell region. By changing the radius of the internal sphere, we modify the CSNC composition. The atomic positions are then optimized. For all the considered systems, electronic properties are calculated within density functional theory (DFT) using the local density approximation (LDA) for the exchange-correlation functional, as implemented in the plane-wave pseudopotential Quantum ESPRESSO (QE)^75,76^ code. A careful analysis of the convergence of both structural and electronic properties, in terms of plane-wave basis set cutoff, has been conducted. Norm-conserving pseudopotentials with a kinetic cutoff for the plane-wave basis set of 40 Ry have been adopted for all the considered systems. NCs have been placed in large cubic cells containing enough vacuum to avoid spurious interactions between replicas. All atomic positions in these supercells have been fully relaxed until the forces acting on each atom were less than 0.003 Ry/a.u. Band-edge properties of the CSNCs have been determined by analyzing the localization of Kohn–Sham (KS) HOMO and LUMO states, as done in previous studies (see for example ref (77)). The effect of spin–orbit coupling (SOC) on the electronic structure has been analyzed and found to be negligible.

It is well-known that the DFT-LDA underestimates the energy gap of semiconductors. However, in the present work, we focus on the calculation of the band-offset properties of CSNCs that depend on the difference in energy between the first Si and Ge state in the conduction and valence band (CB and VB), respectively. By calculating differences between relative energies, we strongly reduce the inaccuracy induced by the LDA.

Furthermore, for one of the smallest NCs (Ge_35_Si_112_H_100_), the band offsets were also estimated within the GW approximation, to check if the DFT-KS band ordering could be affected by many-body effects. Calculations have been performed adopting 80 000 plane waves for the calculation of the exchange part of the self-energy Σ_*x*_ and 30 000 plane waves for both the screening ϵ^–1^ and the correlation part of the self-energy Σ_*c*_. The total number of bands was set to 2500 for ϵ^–1^ and to 3700 for Σ_*c*_.^78^ A spherical cut of the Coulomb interaction was used to avoid spurious interactions between periodic replicas. Noticeably, the results obtained point out that DFT outcomes are not altered by the inclusion of GW corrections: in general, GW corrections act practically in the same way when applied to Si and Ge near edge states,^22^ which confirms the validity of our approach (see Supporting Information).

## 3 Results and Discussion

In the following, we focus our attention on the study of the band-offset properties of SiGe and GeSi CSNCs of different sizes. The section is divided into three parts. In Section 3.1, we discuss the intrinsic properties of Si and Ge materials and we study the band-offset character of SiGe and GeSi CSNCs with diameters ranging from 1.8 to 3.0 nm. In Sections 3.2 and 3.3, we discern the role played by strain and confinement of the electronic charge density on the band-offset properties of the analyzed systems.

### 3.1 Intrinsic Band Alignment and SiGe and GeSi CSNC Band-Offset Properties

As a preliminary step, a simple estimation of the band-offset properties of a semiconductor-semiconductor junction can be obtained starting from the intrinsic properties of the isolated materials, i.e., by evaluating the intrinsic energy band alignment of the corresponding bulk phases. Obviously, when this scheme is adopted, the effects related to the presence of the true interface (for example bonds, strain or defects), are neglected.

The electronic affinities χ of crystalline Si and Ge are χ_*Si*_ = 4.05 eV and χ_*Ge*_ = 4.00 eV, while their energy gaps are *E*_*G*_^*SI*^ = 1.12 eV and *E*_*g*_^*Ge*^=0.66 eV, respectively. As a consequence, the band alignment between Si and Ge bulks leads to an intrinsic type II offset, with the valence band maximum (VBM) localized on Ge (valence band offset, VBO ≈ 0.46 eV) and the conduction band minimum (CBM) localized on Si (conduction band offset, CBO ≈ 0.05 eV). A type II offset was also theoretically predicted within DFT and GW schemes by aligning, with respect to the vacuum level, band-edge energies of H-terminated Si and Ge surfaces.^22^ In this case, the CBO (VBO) was estimated to be 0.08 (0.59) eV. A larger CBO is expected when the energy level alignment is evaluated between isolated Si and Ge nanostructures of the same size. For instance, the DFT energy level alignment of two spherical H-terminated Si and Ge NCs of about 2.8 nm of diameter leads to a CBO of about 0.3 eV, while the VBO is reduced to 0.27 eV. This result is not surprising, because Ge shows a stronger QCE than Si, in the conduction band, upon size reduction.^79,80^ Indeed, it has been both theoretically and experimentally proven that, when reducing the size of the system, the bandgap in semiconductors opens with a fixed ratio of the valence and conduction band edge shift,^80−84^ that is, Δ*E*_*VBM*_^*Si*^ ÷ Δ*E*_*CBM*_^*Si*^ ≈ 2 for Si and Δ*E*_*VBM*_^*Ge*^ ÷ Δ*E*_*CBM*_^*Ge*^ ≈ 1 for Ge.

Band-offset properties of low-dimensional Si/Ge heterostructures, however, cannot be uniquely determined by only considering the intrinsic properties of the Si and Ge materials, that is, by simply aligning (with respect to the vacuum energy) the ionization potential (IP) and electronic affinity (EA) of isolated (nano)materials of similar size.^56^ In particular, for core–shell nanosystems, the different relevance of the QCE between the core and the shell regions, as well as the lattice mismatch of ∼4% between Ge and Si (which introduces strain and thus band alignment modifications), can significantly alter the intrinsic band alignment of the Si/Ge interface.

In order to investigate the mechanisms that influence the band-offset properties of SiGe and GeSi CSNCs, we consider structures with different sizes and compositions. As a first step, starting from small pristine Si and Ge NCs of nearly 1.8 nm of diameter (Si_147_H_100_ and Ge_147_H_100_, respectively), we generate a set of small SiGe and GeSi CSNCs with different ratios of Si and Ge atoms.

**Figure 1:**
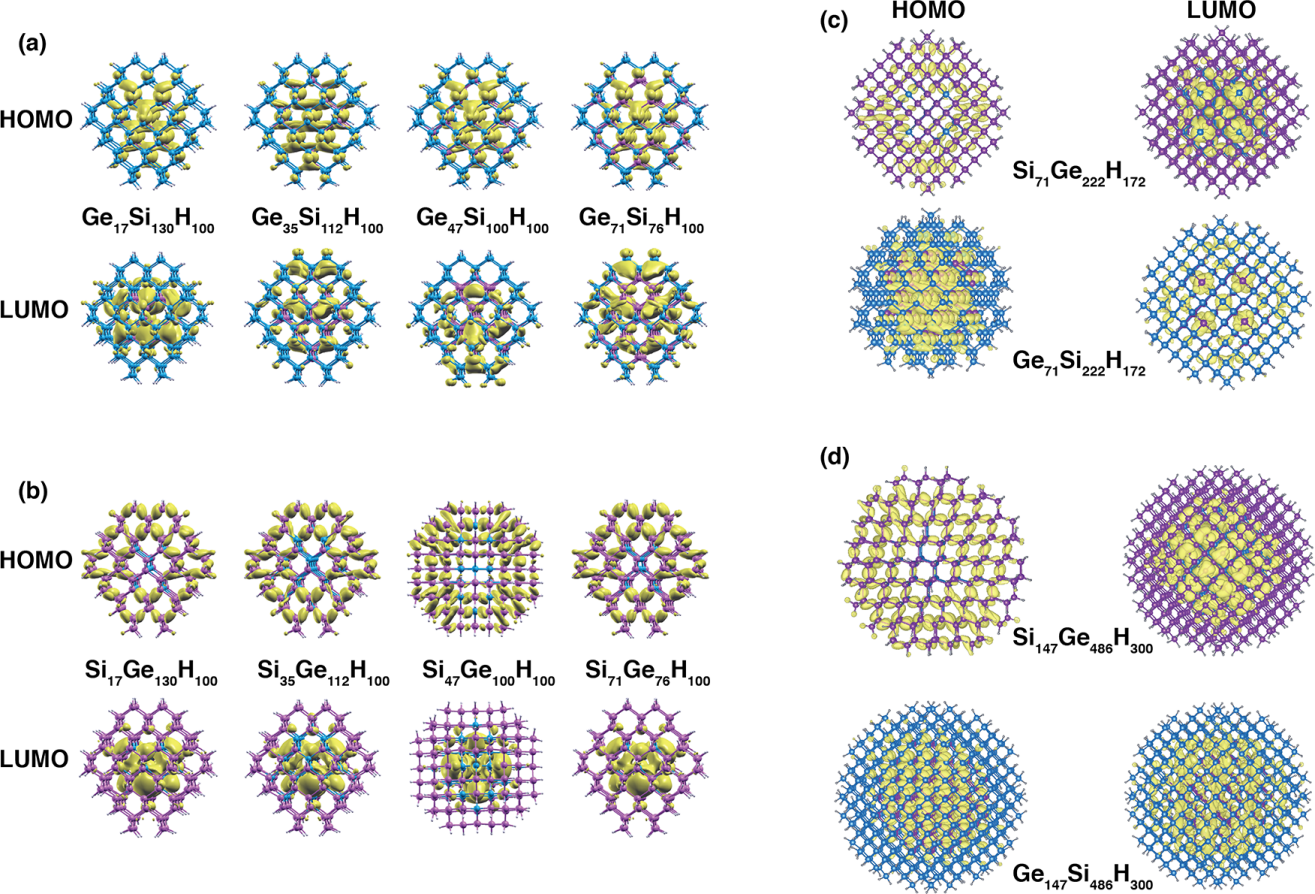
The spatial localization of the electronic wave functions of the HOMO and LUMO states for several CSNCs. (a) GeSi and (b) SiGe CSNCs of diameter of about 1.8 nm. (c) SiGe and GeSi CSNCs with diameter of about 2.4 nm and core diameter of ∼1 nm. (d) SiGe and GeSi CSNCs with diameter of about 3.0 nm and core diameter of about 1.6 nm.

The HOMO and LUMO states localization for SiGe and GeSi CSNCs of about 1.8 nm of diameter is depicted in Figure 1, panels (a) and (b). The two systems manifestly show a different behavior. As for SiGe CSNCs, near-valence-edge states (in particular, the HOMO state depicted in Figure 1, panel b) are mainly localized in the shell region, while near-conduction-edge states (and particularly the LUMO state) are mainly localized in the core region, thus leading to the formation of a type II offset. Noticeably, this behavior is independent of the CSNC composition. On the contrary, in GeSi CSNCs, both the near-valence and near-conduction-edge states (in particular the HOMO and LUMO states, see Figure 1, panel a) are mainly localized in the core region, that is, on Ge atoms. Hence, in this case, the offset presents a type I character. Again, the results are independent of the CSNC composition.

**Figure 2:**
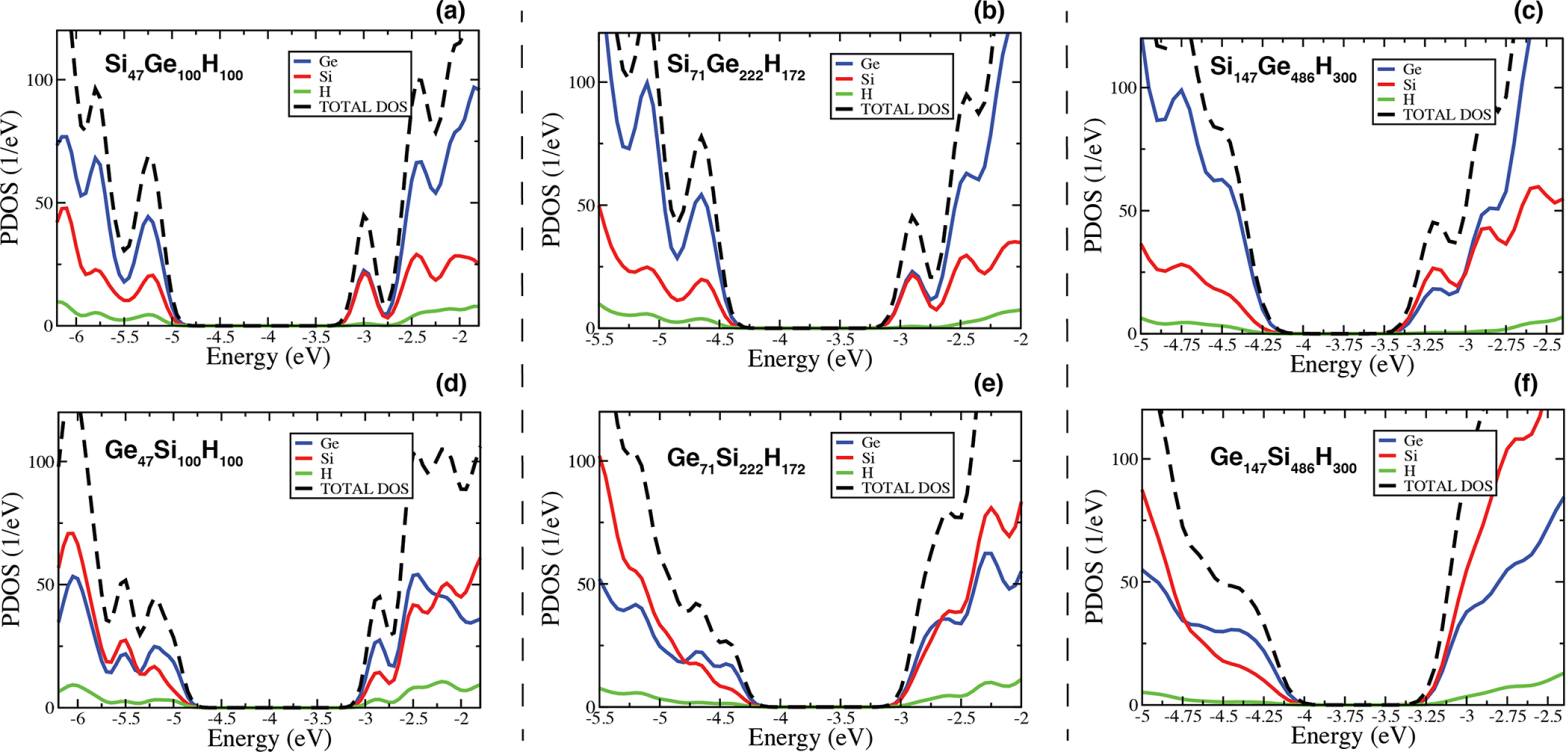
Total density of states (black-dashed line) and PDOS (colored lines) are reported for SiGe and GeSi CSNCs of diameters of 1.8 (a,d), 2.4 (b,e), and 3.0 (c,f) nm. Contributions from Ge atoms (blue-solid line), Si atoms (red-solid lines), and H atoms (green-solid lines) are underlined in the figure.

Similar results are obtained for both CSNCs with a diameter of 2.4 and 3.0 nm. This is shown in the right panel of Figure 1, where the HOMO and LUMO states localization is depicted for the Si_71_Ge_222_H_172_ and the Ge_71_Si_222_H_172_ (panel c) and for the Si_147_Ge_486_H_300_ and Ge_147_Si_486_H_300_ (panel d) (for simplicity, we report only the HOMO and LUMO wave functions localization for CSNCs with a ratio between the number of atoms in the shell and in the core of about 3). In all these cases, SiGe CSNCs show a type II offset, while the band-offset character of the GeSi NCs mainly resembles that of a type I heterostructure, with both the HOMO and LUMO states mainly localized on the Ge atoms, in particular, the LUMO in the outermost part of the core region, near the Si/Ge interface. This is an important point that will be resumed later. Differences between band-offset characters of SiGe and GeSi CSNCs emerge also by the results of Figure 2, where the projected density of states (PDOS) is calculated for several SiGe (panels a–c, Figure 2) and GeSi (panels d–f, Figure 2) CSNCs. For what concerns the GeSi CSNCs, in particular, electronic states near both the valence and the conduction band edges have a clear Ge-like character, which indicates the formation of a type I offset. This Ge-like character, however, decreases moving from the smallest to the largest NC, which suggests a reduction of the CBO with increasing NC size. This behavior is confirmed by the analysis of the spatial localization of the KS unoccupied states. In the smallest GeSi NCs (Ge_47_Si_100_H_100_), indeed, the first unoccupied state localized on the Si (LUMO^*Si*^) is 0.29 eV above the first unoccupied state localized on the Ge (LUMO^*Ge*^), which in this system constitutes the CBM. The CBO is reduced to 0.24 eV when the size is increased (Ge_71_Si_222_H_172_) and moves to only 0.08 eV for the larger NC (Ge_147_Si_486_H_300_). Therefore, the Ge_147_Si_486_H_300_ still shows a type I offset, but in this system, DFT predicts LUMO^*Ge*^ and LUMO^*Si*^ states to be almost degenerate energy levels, meaning that GeSi CSNCs of *d* = 3 nm are close to a type I → type II band-offset transition. The calculation of wave functions localization and of the PDOS are useful to understand band-offset properties of both SiGe and GeSi CSNCs but does not allow (i) to understand which mechanisms lead to the formation of a well-defined offset, (ii) to determine which parameters differentiate the behavior of the SiGe from that of the GeSi, and finally (iii) to explain the trend of the band offset as the size of the NC (and therefore the core and shell thickness) increases. These points will be addressed in the next section. Since the main differences between the SiGe and GeSi CSNCs are related to the localization of the LUMO state, in the following, we will mainly focus our attention on the CBO properties.

### 3.2 SiGe and GeSi Core–Shell NCs: The Role of Strain

Effects induced by strain and QC on both the NC energy gap and the energy of the valence and conduction band edges are often evaluated, starting from the bulk electronic properties, by using a macroscopic model. In this framework, strain and QC are included analytically to correct the energies of the bulk band edges. While this approach has been applied with success to estimate electronic properties of spherical NCs constituted by a single material, its application to CSNCs is more complicated and, in some cases, less predictive. It requires a detailed calculation of the deformation potentials of both valence and conduction bands that have to be specifically obtained for each investigated structure (while often the models implement the deformation potential of the bulk) and a detailed analytical treatment of the QCE that has to consider the role played by both the spherical core region and the shell.

In our approach, we estimate the effects induced by strain and QC following a different procedure, that is, by treating separately the core and the shell regions. Starting from each CSNC, we obtain two different structures. The first one is obtained by deleting the shell atoms. Hence, just the core region of the CSNC remains, and we cap its surface with hydrogen atoms. The second one is obtained by deleting the core atoms. Just the shell region of the CSNC remains, and we passivate with hydrogens all the newly created internal dangling bonds. As a first step, only the additional hydrogen positions are relaxed, keeping both Si and Ge atomic positions unaltered, thus preserving the strain induced by the formation of a Si/Ge interface on both the core and the shell regions. Since, in this case, the Si/Ge interface is not explicitly taken into account, the adopted model cannot exactly reproduce the band-offset properties of SiGe and GeSi CSNCs reported in Figure 1. For instance, by impeding the wave function delocalization on both the core and shell regions, it leads to a slight overestimation of the confinement of the electronic charge density. However, it is a good approximation (i) to clarify the role played by strain or, more generally, by the structural distortions induced by the formation of a Si/Ge interface and (ii) to understand if the QCE has a different relevance in the core and shell regions. By comparing the results obtained considering the entire structures with the ones resulting from this model, where the core and the shell are investigated separately, we have estimated a discrepancy of about 0.1–0.15 eV in both the VBO and the CBO, that represents, as a good approximation, the uncertainty of the method.

**Figure 3:**
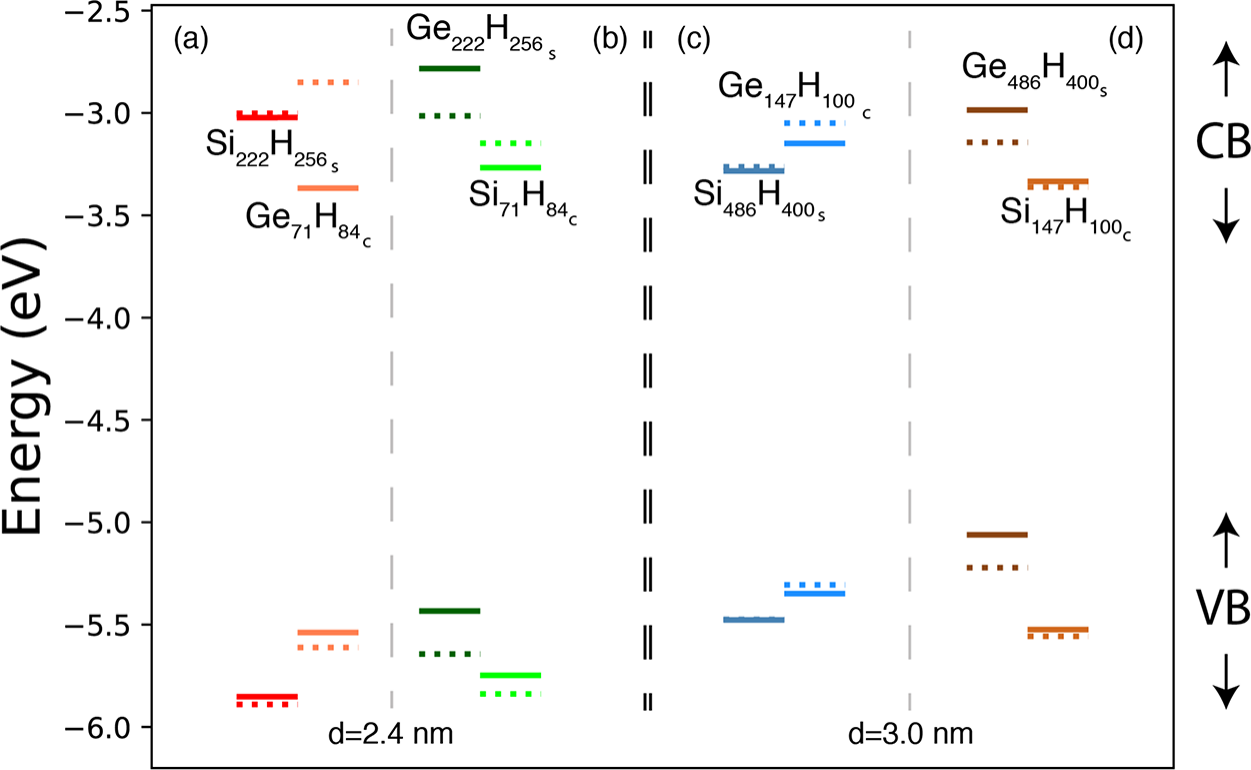
In panels (a) and (b), solid lines refer to the HOMO and LUMO energies calculated with respect to the vacuum level for the core and shell structures extracted from the CSNCs of *d* = 2.4 nm, that is, the Ge_71_Si_222_H_172_ (a) and the Si_71_Ge_222_H_172_ (b). Dotted lines refer to the relaxed structures. In panels (c) and (d), the same analysis is performed considering the systems obtained from the CSNCs of *d* = 3.0 nm, that is, the Ge_147_Si_486_H_300_ (c) and the Si_147_Ge_486_H_300_ (d). The energy scale is obtained through the vacuum level alignment rule where the vacuum energy is set to 0 eV. The labels CB and VB indicate that levels refers to conduction band (CB) or valence band (VB) states.

In Figure 3, solid lines of the panels (a) and (b), we report the energy level alignment calculated for the HOMO and LUMO states for the structures extracted from the GeSi, panel (a), and from the SiGe, panel (b), CSNCs with a diameter of 2.4 nm. The systems are the Si_222_H_256*s*_ and the Ge_71_H_84*c*_ (extracted from the *d* = 2.4 nm GeSi CSNC, panel (a)) and the Ge_222_H_256*s*_ and the Si_71_H_84*c*_ (obtained starting from the *d* = 2.4 nm SiGe CSNC, panel (b)). The labels *c* and *s* indicate that the structure has been obtained from the core and the shell region, respectively. In panels (c) and (d), the same analysis is performed for the nanostructures extracted from the GeSi (the Si_486_H_400*s*_ and the Ge_147_H_100*c*_) and SiGe (the Ge_486_H_400*s*_ and the Si_147_H_100*c*_) CSNCs with a diameter of 3.0 nm.

For what concerns the results obtained for the structures extracted from the *d* = 2.4 nm CSNCs, the model predicts a type I offset for the GeSi structure (CBO = 0.35 eV) and a type II offset for the SiGe system (CBO = 0.48 eV) in good agreement with the outcome of Section 3.1. For what concerns the structures extracted from the *d* = 3.0 nm CSNCs, the model correctly predicts a type II character for the SiGe CSNC with a CBO of about 0.35 eV, while it is not able to predict a type I offset for the *d* = 3.0 nm GeSi CSNC. This is not surprising, because the Ge_147_Si_486_H_300_ shows a type I offset with a CBO of only 0.08 eV (the LUMO^*Ge*^ and the LUMO^*Si*^ are almost degenerate), that is, their separation in energy is lower than the accuracy of the procedure here adopted. In Figure 3, dotted lines refer to the HOMO and LUMO energy levels of the fully relaxed structures where the strain induced by the presence of a Si/Ge interface has been removed. A comparison between solid and dotted lines will help to clarify the role played by strain.

Obviously, strain mainly affects the electronic properties of the smaller NCs, as clearly underlined by the results reported in Figure 3, where the separations in energy between dotted and solid lines is more marked for the systems reported in panels (a) and (b). Moreover, it mainly impacts the energy of the LUMO^*Ge*^ state, while its effect on the energy of the LUMO^*Si*^ state is generally less relevant. This condition is clearly underlined, for instance, by the energy diagram of Figure 3, panel (a), which points out a reduction of 0.51 eV of the EA of the Ge_71_H_84*c*_ (that is, a shift toward higher energies of the LUMO^*Ge*^) when we move from the strained to the unstrained structure (from solid to dotted orange lines, panel a, CB states). At the contrary, the LUMO^*Si*^ of the Si_222_H_256*s*_ is practically unaffected by lattice relaxation. From Figure 3, panel (a), we observe that the energy level lineup obtained considering the strained systems show a type I character (solid lines), while the unstrained configurations lead to a type II band-offset (dotted lines). This is a fundamental result, because it points out that strain is responsible for the formation of a type I offset in small GeSi CSNCs. We now move to analyze the results depicted in Figure 3, panel (b), where the HOMO and LUMO energies of the structures extracted from the *d* = 2.4 nm SiGe CSNC are reported. Noticeably, the different dislocations of the Si and Ge atoms, the Si in the core and the Ge in the shell, imply a different response to strain. In this case, indeed, we observe a reduction of the energy of the LUMO^*Ge*^ (that is, an increment of the EA) moving from the strained to the unstrained system (from solid to dotted dark green lines, panel (b), CB states) associated with a light increment of the LUMO^Si^ energy (from solid to dotted light green lines, panel (b), CB states). As a consequence, in this case, strain strengthens the intrinsic type II character of the SiGe interface, leading to an increment of the CBO with respect the unstrained systems.

Similar trends are observed for the systems extracted from the *d* = 3.0 nm GeSi and SiGe CSNCs (Figure 3, panels (c) and (d), respectively); in this case, however, the effects induced by strain are less relevant due to the larger size of both core and the shell.

**Figure 4:**
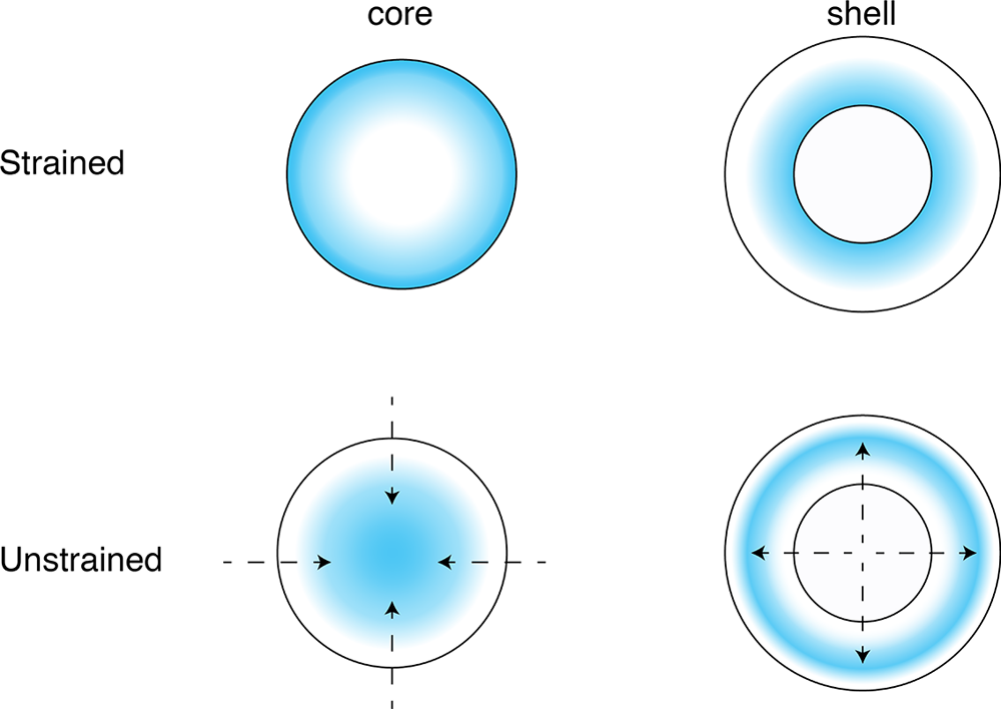
The localization of the LUMO^Ge^ is schematized in the figure for both the strained and the unstrained system. In the strained system, LUMO^Ge^ is localized in proximity of the Si/Ge interface, that is, in the outermost part of the core or in proximity of the internal part of the shell, depending on the Ge localization in the core or in the shell. When strain is removed, the LUMO^Ge^ appears to be more confined in the core region or less confined in the shell region depending on the Ge occupies the core or the shell, respectively. The arrows help in understanding how the LUMO^Ge^ localization changes when we move for the strained to the unstrained configuration.

In order to interpret the results reported in Figure 3, we have calculated the localization of the LUMO wave functions for both the strained and the unstrained systems. For what concern the LUMO^Ge^, we have observed that, for the “strained” systems, this state is always mainly localized in the proximity of the Si/Ge interface. In particular, the LUMO^Ge^ is mainly located in the outermost part (internal surface) of the core (shell) when this region is occupied by Ge atoms. When strain is removed, we always observe a change in the LUMO^*Ge*^ localization, which moves away from the Si/Ge interface and appears to be more localized in the central part of the region occupied by the Ge. This behavior is schematized in Figure 4. For the system where Ge is in the core, therefore, the removal of strain implies a greater confinement of the LUMO^*Ge*^, with a consequent shift of the level toward higher energies, as observed in Figure 3. At the contrary, when Ge is localized in the shell, strain removal produces a reduction of the confinement of the LUMO^*Ge*^, which is now distributed on a larger volume, implying a lowering of its energy. Strain is therefore responsible for a change of the LUMO^*Ge*^ confinement, acting differently depending on the Ge localization in the core or in the shell. The effects above-described occur also for the LUMO^*Si*^, but in this case, the changes in the wave function localization are less marked. Moreover, the LUMO^*Si*^ is less sensitive than the LUMO^*Ge*^ to changes in the wave function confinement, which explains the minor effects induced by strain on the LUMO^*Si*^ energy. In other words, the Si is less sensitive to strain than the Ge, as also pointed out in ref (85). The outcomes of this section clarify that a first condition to be verified in order to observe a type II offset in the GeSi CSNCs is that the core has to be sufficiently large to reduce the effects induced by strain. When this is not verified, structural distortions present in the core region (induced by the Si and Ge lattice mismatch) induce a lowering of the LUMO^*Ge*^ energy and thus a type I offset.

In addition to the strain, also the QCE can alter the intrinsic band alignment of the Si/Ge interface, especially when small CSNCs are take into account. We will discuss this point in the next section.

### 3.3 SiGe and GeSi Core–Shell NCs: The Role of Quantum Confinement

**Figure 5:**
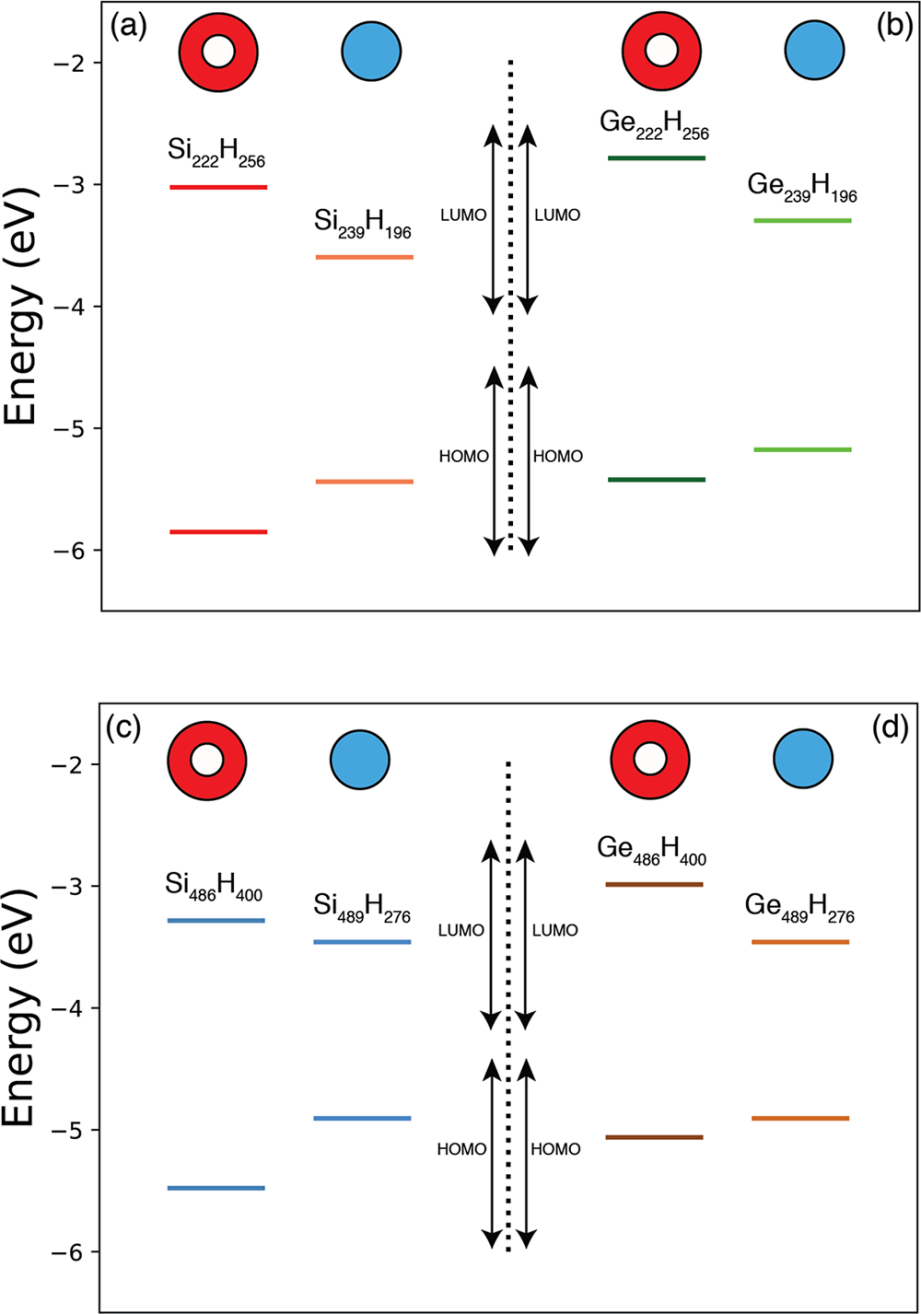
The HOMO and LUMO energies calculated for the nanostructured spherical caps Si_222_H_256*s*_ and Ge_222_H_256*s*_ (Si_486_H_400*s*_ and Ge_486_H_400*s*_) and for the spherical Si_239_H_196_ and Ge_239_H_196_ (Si_489_H_276_ and Ge_489_H_276_) NCs are reported in panels (a) and (b) (panels (c) and (d)). Different colors identify different structures. The energy scale is obtained through the vacuum level alignment rule where the vacuum energy is set to 0 eV.

Results of Figure 3 are also affected by the QCE. We have previously underlined that LUMO^*Ge*^ is generally more affected by QC than the LUMO^*Si*^. However, in a CSNC, the core and the shell have different shapes (a nanosphere for the core and a nanostructured shell cap for the shell) and extensions, and therefore, the effects induced by QC on a well-defined material could be different depending on the material occupying the core or the shell region. This is a very important point that has to be addressed in order to comprehend the microscopic properties of GeSi and SiGe CSNCs. We focus our attention on the behavior of the LUMO levels, since the VBO shows, for all the considered CSNCs, the same character as the intrinsic Si/Ge heterostructures. In order to understand how QC depends on the nanostructure shape, we consider the systems obtained by extracting the shell region from the CSNCs, i.e., the nanostructured shell caps Si_222_H_256*s*_, Ge_222_H_256*s*_, Si_486_H_400*s*_, and Ge_486_H_400*s*_, and we compare their electronic properties with the ones of H-terminated spherical NCs containing a similar number of Si or Ge atoms, i.e., the Si_239_H_196_, the Ge_239_H_196_, the Si_489_H_276_, and the Ge_489_H_276_ NCs. This will help us to clarify in terms of charge density confinement, what happens when the same number of atoms is distributed in the core or in the shell, that is, in which region QC is generally more relevant. The obtained results are reported in Figure 5, where the energies of the HOMO and LUMO states of the nanostructured shell caps and spherical NCs are reported with respect to the vacuum energy. We can observe that both energy gaps and IP (measured as the energy distance between the HOMO and the vacuum level) are larger in the nanostructured shell caps than in the corresponding spherical NCs, while the EA (measured as the energy distance between the LUMO and the vacuum level) is smaller. For instance, the EA of the Si_222_H_256*s*_ is, in absolute value, 0.57 eV lower than the one of the Si_239_H_196_, while the EA of the Si_486_H_400*s*_ is 0.18 eV lower than the one of the Si_489_H_276_. Considering that strain does not essentially affect the energy of the LUMO^*Si*^, we can safely affirm that the QCE is more pronounced when Si atoms are distributed in a shell cap than inside a sphere. Regarding Ge nanostructures, we find that the calculated EA for the Ge_222_H_256*s*_ is, in module, 0.51 eV lower than the one of the Ge_239_H_196_ and that the EA obtained for the Ge_486_H_400*s*_ is 0.47 eV lower than the one of the Ge_489_H_276_. Even for Ge, therefore, we can say that the QCE is more pronounced when atoms are distributed in a shell cap than inside a sphere, and this is true when we consider both strained and fully relaxed systems.

We see that, for reasons related to the geometry of the system, QC acts differently in the core and in the shell regions of CSNCs and therefore, the band-offset character of SiGe and GeSi CSNCs cannot be, in principle, determined by simply considering the band energy alignment of Si and Ge bulks, surfaces or NCs of the same size.

In GeSi CSNCs, two different effects, both related to QC, concur with strain in determining the band-offset character of the system. The first one, already discussed in above and in Section 3.1, implies that, in similar Si and Ge nanostructures, QC acts more markedly on the LUMO^*Ge*^ than on the LUMO^*Si*^, with a consequent shift to higher energies of the LUMO^*Ge*^, and a reinforcement of the type II offset. The latter, in competition with the former one, implies that the electronic charge confinement is generally more relevant in the shell than in the core region, leading to a more important shift of the LUMO^*Si*^ to higher energies than the LUMO^*Ge*^, a condition that favors the formation of a type I offset. In GeSi CSNCs with a thin shell (less that ∼0.7 nm), the second effect can be dominant and foster the formation of a type I offset. Obviously, the larger the shell, the weaker QC is in this region. The two aforementioned effects are instead combined to enhance the shift of the LUMO^*Ge*^ toward higher energies when the SiGe CSNCs are considered. In this case, the different relevance of the QCE in the core and shell regions strengthens the formation of a type II offset.

The obtained results point out that two conditions have to be verified in order to observe a type II offset in GeSi CSNCs. In particular, (i) the core has to be sufficiently large (more than ∼1.5 nm of diameter) in order to reduce the effects induced by strain on the LUMO^Ge^ level, and at the same time, (ii) the shell has to be sufficiently large (thickness more than ∼0.7 nm) in order to reduce the effects induced by QC on the LUMO^Si^. Conditions (i) and (ii) cannot, in general, be simultaneously verified in small GeSi CSNCs, approximately with diameter smaller than 3 nm. For instance, the Ge_71_Si_562_H_300_ NC with *d* = 3 nm, still shows a type I offset with a CBO of 0.13 eV. With respect to the Ge_147_Si_486_H_300_ depicted in Figure 1, this system shows a larger shell (and thus a reduced QC in this region) and a smaller core (and thus more pronounced effects induced by strain in this region), which imply a simultaneous lowering of both the LUMO^*Si*^ and LUMO^*Ge*^ energies with respect the ones of the Ge_147_Si_486_H_300_ (the former induced by the slight reduction of the QCE in the shell region, the latter by increasing strain in the core region), which would not produce changes in the band-offset character.

**Figure 6:**
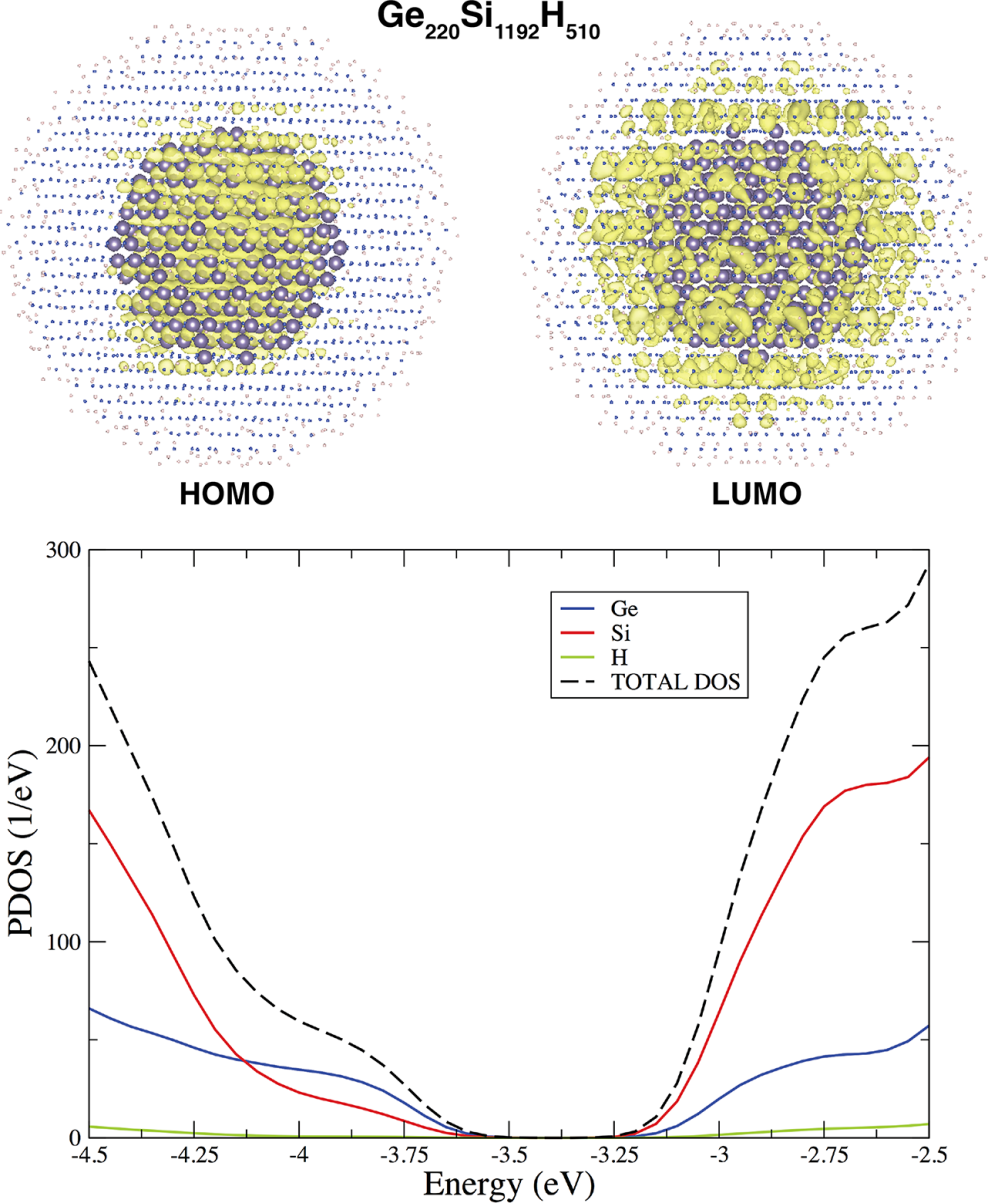
In the top panel, we report the localization of the HOMO and LUMO states for the Ge_220_Si_1192_H_510_ CSNC. To make more visible the localization of these states, both H and Si atoms have been shrunk. In the bottom panel, calculated PDOS is reported. Both panels clearly indicate the formation of a type II offset.

The diameter *d* = 3 nm represents a sort of critical size for the GeSi CSNCs. Below this threshold, we clearly observe a type I offset with both the HOMO and LUMO states mainly localized on the Ge atoms; the same offset is observed for GeSi CSNCs with a diameter of about 3 nm, but in this case, we are in proximity of the type I → type II transition. Finally, a type II offset can result for GeSi CSNCs with *d* > 3 nm. This condition guarantees the possibility of obtaining CSNCs with, at the same time, a sufficiently large shell to reduce the QCE in the Si region and a sufficiently large core to reduce the effects induced by strain in the Ge region. As an example, we report in Figure 6 the case concerning the Ge_220_Si_1192_H_510_ (*d* ≈ 4 nm, *d*_*core*_ ≈ 2 nm) which shows a type II band offset, with the HOMO mainly localized in the core around the Ge atoms, and the LUMO localized on the Si atoms, thus outside of the core region, in between the Si/Ge interface and the outermost part of the shell. The formation of a type II band offset is also confirmed by the calculated PDOS, as reported in Figure 6, bottom panel.

## 4 Conclusions

In this paper, density functional theory has been adopted in order to investigate the mechanisms involved in the formation of the band offset of SiGe and GeSi CSNCs and, in particular, to discern the role played by strain and quantum confinement. NCs with diameters ranging from 1.8 to 4.0 nm have been considered. This analysis is crucial because, depending on the band-offset character (type I or type II), NCs are more suitable to be engineered in light-emitting (type I) or in photovoltaic (type II) devices. We show that both strain and QC contribute to the formation of type II offset in SiGe CSNCs, with the HOMO localized in the Ge shell and the LUMO localized in the Si core. In these systems, therefore, the band-edge properties resemble those obtained by simply considering the intrinsic properties of the Si and Ge materials. The analysis is far more subtle for GeSi CSNCs. In these NCs, the HOMO state is always localized in the core region, while the LUMO localization depends on the geometry of the system, which determines the relevance of strain and QC on both core and shell regions. Our results point out that, when the Ge is in the core, the strain contributes to lower the energy of the LUMO^*Ge*^ state, thus favoring the formation of a type I offset. This effect is related to a change in the LUMO^*Ge*^ confinement induced by strain. Moreover, the QCE is generally more pronounced in the shell than in the core region and, in GeSi CSNCs, it contributes to move the LUMO^*Si*^ level to higher energies. As a result, in GeSi CSNCs with a diameter less than 3 nm, the LUMO is always localized on the Ge and the band offset shows a type I character. Depending on the band-offset properties, spherical GeSi CSNCs can be grouped into three different classes: (i) CSNCs with a diameter less than 3 nm, which are clearly characterized by a type I offset; (ii) CSNCs with a diameter of about *d* = 3 nm, which still show a type I offset, although they have a critical size for observing a type I → type II transition; (iii) CSNCs with diameters larger than *d* = 3 nm, which can present a type II offset, because, in this case, the core and the shell are sufficiently large to reduce the effects induced by strain in the internal core region, and by QC in the external shell. This has been directly demonstrated in the case of a large Ge_220_Si_1192_H_510_ CSNC, where calculations reveal indeed a type II band offset.

## Supporting Information Available

Results concerning the electronic gaps, the TDDFT spectra, and quasiparticle (GW) effects on the electronic gap (PDF)
