# Supplementary material for: Interplay of Quantum Confinement and Strain Effects in Type I to Type II Transition in GeSi Core–Shell Nanocrystals
Source: J Phys Chem C Nanomater Interfaces. 2023 Jan 5;127(2):1209–19. doi: 10.1021/acs.jpcc.2c07024 (PMC9869394; doi:10.1021/acs.jpcc.2c07024)
Supplement: Supplementary file 1 — jp2c07024_si_001.pdf [file jp2c07024_si_001.pdf]

Supporting Information of "Interplay of Quantum Confinement and Strain Effects in Type I to Type II Transition in GeSi Core-Shell Nanocrystals"

Ivan Marri,[*,†,‡,¶] Simone Grillo,[§] Michele Amato,[‖] Stefano Ossicini,[†,¶,⊥] and Olivia Pulci[§]

†*Department of Sciences and Methods for Engineering, University of Modena and Reggio Emilia, 42122 Reggio Emilia, Italy*

‡*Interdepartmental Center for Research and Services in the Field of Hydrogen Production, Storage and Use H2 – MO.RE, Via Università 4, 41121 Modena, Italy*

¶*Centro Interdipartimentale En&Tech, 42122 Reggio Emilia, Italy*

§*Department of Physics, University of Rome Tor Vergata, and INFN, Via della Ricerca Scientifica 1, I-00133 Rome, Italy*

‖*Université Paris-Saclay, CNRS, Laboratoire de Physique des Solides, 91405, Orsay, France*

⊥*Centro S3, Institute of Nanoscience — Italian National Research Council (CNR-NANO), via Campi 213/A, 41125 Modena, Italy*

E-mail: marri@unimore.it

In this section, we report additional information concerning electronic and optical properties of the systems analysed in the manuscript. Calculated LDA energy gaps E_{gap}^{LDA} are reported in Table S1. It is evident that the E_{gap}^{LDA} depends only marginally on the CSNCs composition and, following the typical trend imposed by the QCE, decreases when the NCs size increases. Moreover, by focusing on the smaller NCs, we can also observe that the

E_{gap}^{LDA} calculated for the CSNCs does not strongly differ from those of the pristine $Si_{147}H_{100}$ and $Ge_{147}H_{100}$ NCs.

For the smaller nanocrystals, we have also calculated the absorption spectra using the Liouville–Lanczos approach to Time-Dependent Density Functional Perturbation Theory, as implemented in the TDDFT tool of the QE package[1]. The results obtained for the pristine $Si_{147}H_{100}$ and $Ge_{147}H_{100}$ NCs and the $Ge_{35}Si_{122}H_{100}$ and $Si_{35}Ge_{122}H_{100}$ CSNCs are depicted in Fig. S1. The spectra calculated for the CSNCs, and their absorption energy thresholds, fall in-between the ones obtained for the $Si_{147}H_{100}$ and the $Ge_{147}H_{100}$ NCs.

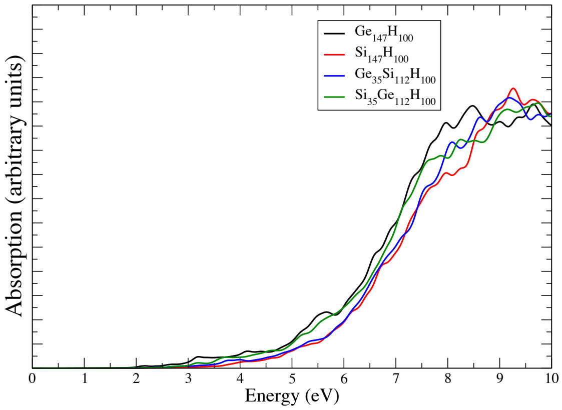

Figure S1: (Color online) Absorption spectra, calculated for the $Ge_{35}Si_{122}H_{100}$ and the $Si_{35}Ge_{122}H_{100}$ NCs are reported in the figure and compared with the ones obtained for the $Si_{147}H_{100}$ and the $Ge_{147}H_{100}$.

In Table S1, we also report the GW gap of one of the smallest NCs, i.e. $Ge_{35}Si_{122}H_{100}$. Quasi-particle calculations were introduced in order to check if a different energy levels alignment could arise when many-body effects are taken into account. Convergence tests are shown in Fig. S2. GW corrections are sizeable, opening the DFT gap by about 1.4 eV; nevertheless, no change in the band-edge ordering is observed. In both DFT and GW, the states around the gap are mainly localized on the Ge core, hence giving a type I heterostructure. The first unoccupied state mainly localized on Si is at 0.29 eV above the LUMO in DFT (0.2 eV above the LUMO in GW); analogously, the first occupied Si state is located 0.46 eV below the HOMO (1.0 eV below the HOMO in GW). Hence, the DFT alignment is confirmed also by quasi-particle calculations.

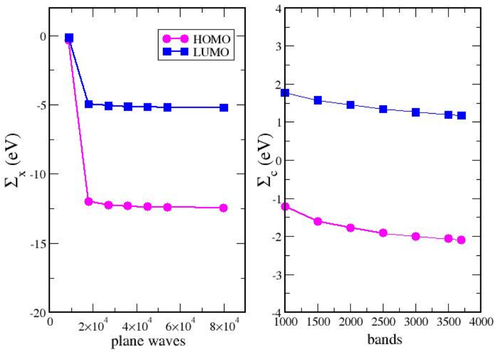

Figure S2: (Color online) Convergence tests for the Self Energy $\Sigma = \Sigma_x + \Sigma_c$ calculated for the HOMO and LUMO levels of the $Ge_{35}Si_{122}H_{100}$ NC. The correlation part of the self-energy Σ_c was evaluated using 30000 plane waves.

Table S1: DFT-LDA energy gaps calculated for GeSi and SiGe CSNCs of different compositions and size are reported in the table. As a reference, for the smaller NCs, the pristine $Ge_{147}H_{100}$ and $Si_{147}H_{100}$ show an energy gap of 2.25 and 2.19 eV, respectively. For what concerns the $Ge_{35}Si_{122}H_{100}$, we have also calculated the GW energy gap which settles to 3.5 eV.

**diameter (nm)**	**Ge(core)/Si(shell)**	**Energy Gap (eV)**	**Si(core)/Ge(shell)**	**Energy Gap (eV)**
1.8	$Ge_{17}Si_{130}H_{100}$	2.09	$Si_{17}Ge_{130}H_{100}$	2.10
1.8	$Ge_{35}Si_{122}H_{100}$	2.12	$Si_{35}Ge_{122}H_{100}$	2.12
1.8	$Ge_{47}Si_{100}H_{100}$	2.11	$Si_{47}Ge_{100}H_{100}$	2.12
1.8	$Ge_{71}Si_{76}H_{100}$	2.16	$Si_{71}Ge_{76}H_{100}$	2.10
2.4	$Ge_{71}Si_{222}H_{1772}$	1.54	$Si_{71}Ge_{222}H_{172}$	1.67
3.0	$Ge_{147}Si_{486}H_{300}$	1.19	$Si_{147}Ge_{486}H_{300}$	1.19
3.0	$Ge_{71}Si_{562}H_{300}$	1.17	-	-
4.0	$Ge_{220}Si_{1192}H_{510}$	0.83	-	-

Notes and references

(1) Malcıoğlu, O. B.; Gebauer, R.; Rocca, D.; Baroni, S. turboTDDFT – A code for the simulation of molecular spectra using the Liouville–Lanczos approach to time-dependent density-functional perturbation theory. *Computer Physics Communications* **2011**, *182*, 1744–1754.
